# West London Post-Graduate College

**Published:** 1907-09-07

**Authors:** 

**Affiliations:** Honorary Physician St. Marylebone General Dispensary


### Graduates' Medical Schools.

### WEST LONDON POST-GRADUATE COLLEGE.

By COLONEL CLUTTERBUCK, M.D., B.S., M.R.C.P., Honorary Physician St. Marylebone General Dispensary.

The West London Hospital, Hammersmith, was the first of the general hospitals in London to reserve its practice strictly for qualified men, and to establish a post-graduate college as an integral part of its constitution: the fame of the College has undoubtedly spread to corners of the earth where, but for its existence, the hospital would have remained unknown: the child has, in a sense, overshadowed the parent. As one who has been intimately connected w[illegible] the college and hospital for many years, and who has had perhaps more experience of teachers in the medical world than usually falls to qualified men, I feel justified in expressing my deliberate opinion that the College still retains the pride of place among similar institutions which it necessarily, from priority of inception, occupied at the commencement of its career. Nor is there any reason for supposing that its utility will not be further increased. Facilities for post-graduate medical instruction are now being largely introduced in the provincial centres, and I feel sure many valuable hints might be obtained by bodies contemplating such institutions from acquaintance with the principles and methods pursued at Hammersmith: while the provincial man who has not as many opportunities as he might wish for visits of inquiry to the metropolis, will carry away many hints and much information, if he is enabled at any time to put in a spell of work there.

Almost the first thing that strikes one on looking over a recent prospectus is the variety of the topics dealt with in one session's work. Our needs and interests are not all alike, but in whatever direction they may lie I may safely say they will be found fully catered for here.

The hospital itself contains 159 beds, and the advantages of the direct connection of the college with the wards and out-patient work are too obvious to need even a reference, but some special features may be emphasised. As regards surgery, for instance, the staff are willing and ready to make use of the assistance of post-graduates in performing operations; the advantage of this privilege is very great to men who perhaps have not many opportunities for dealing with major operations, but who nevertheless occupy positions where they may be at any moment called upon to undertake them. Especially does this apply to the large and important branch of abdominal surgery, which has been revolutionised within the past few years. New operations and treatments are, of course, practised.

I may here mention that the hospital is recognised by the Royal College of Surgeons as an institution where candidates (who need not be Members) for the Fellowship can spend the necessary year of study after obtaining a registrable qualification, while the college and hospital are also recognised by the naval and military authorities as regards study leave.

Members of the college may also accompany the resident staff on their visits to the wards, and so have opportunities of refreshing their recollections of such procedures as lumbar puncture, with cyto-diagnosis, and gastric lavage, with chemical and microscopical examination of gastric contents. In the theatre post-graduates may, under the direct supervision of the anæsthetists of the staff, administer anæsthetics in major operations; most men would prefer to try a new anæsthetic, as, for instance, ethyl-chloride, under such conditions, rather than by themselves from merely book knowledge. The effect and manner of employment of the ever-increasing number of drugs used for producing local anæsthesia can also be studied to advantage.

The out-patient departments necessarily resemble in their main features those of other general hospitals, and the staff are always alert to discover, and, when found, to demonstrate, any cases of exceptional interest, or, what is of equal importance, any points of special interest found in cases of the more common diseases. There is one branch of out-patient practice where even the numbers of themselves constitute a valuable element in instruction. I refer to skin diseases. Everyone knows how difficult minor differences in persons suffering from the same condition sometimes make diagnosis, and those who are not satisfied to put every condition to which they cannot at once give a specific name into the rubbish heap of eczema will appreciate the value of the mere multiplication of cases with the advantage of having them clearly expounded by an expert. Another such branch is that of the infants' and children's clinic: if any fault is to be found here it is that it is too large, leaving an impression that one could profitably spend more time in examining fewer cases. The increasing attention paid to pædiatrics makes it difficult to give up too much time to this study; for it is almost an axiom that in general practice a competent and sympathetic treatment of children is one of the broad roads to success. In the out-patient clinics post-graduates are asked questions, if they wish; a valuable form of instruction for men intending to sit for examinations. All cases may be fully examined in the rooms provided for the purpose. In the gynæcological department, too, examinations necessarily restricted in private practice are part of the routine work. In all these departments clinical assistants are appointed from post-graduates desiring to hold such positions. The electrical department is fully equipped for diagnosis and treatment; an *x*-ray department and baths are also provided. Leading off the out-patient rooms is a dark room for ophthalmoscopic and laryngoscopic work. All through the year lectures are given daily, except Saturdays, in the afternoons, on practical medicine and surgery in all their branches. There are also short courses on tropical diseases, and on mental diseases by men who have made their mark in those subjects. These lectures are quite distinct from the clinical demonstrations in the wards by the physicians and surgeons, and may be attended without any additional fee. I would also mention the pathological department. The specimens are specially selected for their value from an instructional point of view, and the demonstrations of naked-eye and microscopical specimens are very thoroughly carried out.

A prominent feature in the arrangements of the College is the provision made for instruction of small classes in every branch of practical medicine. Such classes are formed every session in connection with diseases of special regions, such as eye, throat, skin, etc.; for the clinical examination of blood and urine; in bacteriology and medical microscopy generally; in applied anatomy, administration of anæsthetics, intestinal surgery, cystoscopy, *x*-ray work, etc. For these classes, each of which is strictly limited to a small number, additional fees are required, as they are practically individual and tutorial. The ordinary fee for membership covers attendance at all general lectures and at all the routine practice of the hospital, whether in the general or special wards or departments. Autopsies can, of course, be attended. Arrangements can be made through the College authorities for special coaching should it be desired.

The West London Medico-Chirurgical Society holds its meetings in the hospital. The subscription is very small, and there is no entrance fee; post-graduates are invited to become members; a quarterly medical journal is sent free to each member; in it are published lists of gentlemen attending the hospital and other college news.

Rooms are provided for members where they may read, write, and smoke, and consult books of reference; tea can be obtained in the reading-room; lockers are provided at a small rent for those who wish to keep books or such articles at the College. The medical library of the West London Medico-Chirurgical Society is housed in the College, and post-graduates have the use of the books and periodicals; for a small subscription they can borrow and take home books; any medical book not in the library can be obtained in 24 hours; in addition lay papers and journals are supplied, the cost of which those using the rooms are asked to help to defray.

The hospital is very accessible; it is within two or three minutes' walk from stations on the District and Metropolitan Railways and the Piccadilly Tube; the terminus of the Central London Railway (Twopenny Tube) at Shepherd's Bush is about twelve minutes' walk from it. In the other direction Barnes, Chiswick, and Kew are all within easy reach. Good lodgings are obtainable in the neighbourhood, a list of addresses being kept by the Secretary of the College. An annual dinner is held of past and present members of the hospital and college, and an annual conversazione is given by the staff, at which all gentlemen who have been connected with the College are welcomed.

We live, however, in a commercial age, and some of us are apt, perhaps too apt, to appraise the value of a thing by its admitted monetary value; it happens that an indirect test of the latter may be legitimately applied in this case. Members may join for any period from one week up to the term of their natural lives, the fees ranging from one guinea to £25. Some few years ago it was considered expedient to increase the fee for life membership, and this enhancement was carried out and remains in force without any resulting diminution of membership. I believe it is proposed, when circumstances are favourable, to enlarge the existing institution by building additional premises, so the College may be expected to have an extended sphere of usefulness.

